# Hydrolyzed Yeast Supplementation in Calf Starter Promotes Innate Immune Responses in Holstein Calves under Weaning Stress Condition

**DOI:** 10.3390/ani10091468

**Published:** 2020-08-21

**Authors:** Eun Tae Kim, Hyo Gun Lee, Dong Hyeon Kim, Jun Kyu Son, Byeong-Woo Kim, Sang Seok Joo, Da Som Park, Yei Ju Park, Se Young Lee, Myung Hoo Kim

**Affiliations:** 1 Dairy Science Division, National Institute of Animal Science, Rural Development Administration, Cheonan 31000, Korea; etkim77@korea.kr (E.T.K.); kimdh3465@korea.kr (D.H.K.); junkyuson@korea.kr (J.K.S.); 2 Department of Animal Science, College of Natural Resources & Life Science, Pusan National University, Miryang 50463, Korea; ggabulzima@naver.com (H.G.L.); kimbw@pusan.ac.kr (B.-W.K.); ssjoo7680@gmail.com (S.S.J.); parkdasome@gmail.com (D.S.P.); zx7777777@pusan.ac.kr (Y.J.P.); 3 Division of Animal Husbandry, Yonam College, Cheonan 31005, Korea; sylee@yonam.ac.kr

**Keywords:** Holstein, calf starter, hydrolyzed yeast, weaning, immune responses

## Abstract

**Simple Summary:**

New-born Holstein calves are vulnerable to infectious diseases owing to incomplete immune system development. This study aimed to evaluate the effects of six weeks of hydrolyzed yeast supplementation in Holstein calf starter on stress and immune responses after weaning. Hydrolyzed yeast supplementation facilitated the innate immune response after weaning, with a limited stress response. Therefore, the supplementation of hydrolyzed yeast in calf starters may help weaned calves adapt to diet-induced stresses. These results suggest that hydrolyzed yeast may be an effective feed additive for modulating stress-related immune responses.

**Abstract:**

Weaned calves are susceptible to infectious diseases because of the stress and malnutrition that occurs during weaning. Therefore, the dairy industry requires effective feed additives to ameliorate stress responses and promote immunity. This study aimed to investigate the effects of hydrolyzed yeast (HY) supplementation on the growth performance, immune and stress parameters, and health status of calves after weaning. Eighteen Holstein calves were randomly assigned to two groups, either receiving a control calf starter or 0.2% HY calf starter from one week of age. All calves were weaned at six weeks of age as a stress challenge. The HY-fed calves had a significantly-higher body weight gain during the post-weaning period (kg/week) compared to the control. Cortisol levels at three days post-weaning (DPW) were significantly lower in the HY group than the control group. Calves fed HY had significantly-higher serum levels of tumor necrosis factor-α and interleukin-1β at one DPW. The HY-fed calves also had higher concentrations of the acute-phase proteins, haptoglobin, serum amyloid A, and transferrin at one DPW. In addition, the diarrhea severity in HY-fed calves was milder after weaning compared to the control group. Our results indicate that HY supplementation reduces stress responses and may promote innate immunity in newly-weaned calves.

## 1. Introduction

The early growth and health status of calves have a significant effect on milk production during the first lactation [1,2]. Several stressors negatively affect the growth and health status of neonatal calves through multiple physiological pathways [3]. Therefore, it is necessary to reduce stress during the calf-rearing period to improve animal welfare and increase profitability. Neonatal calves are particularly vulnerable to infectious diseases as their immune systems have not fully developed [4,5,6]. Cortisol is a hormone released by the adrenal cortex; it is generally known as a stress biomarker and dampens immunity [7]. The weaning process increases the serum levels of cortisol in dairy calves [8,9], indicating that weaning is a major stressor. Furthermore, weaning weakens the immune system through reductions in pro-inflammatory responses, neutrophil trafficking, and phagocytic functioning [4,10,11]. A weakened immune system results in a high level of infectious diseases in neonatal calves after weaning [12].

The administration of dietary immune modulators to weaned calves is a promising approach for improving animal health and preventing a reduction in immunocompetence. There are several dietary immune modulators available in the dairy industry, including yeast and its derivatives (e.g., yeast cell wall products). Yeast and its derivatives are used as supplements for beef and dairy cattle and may improve the growth performance and metabolism of ruminants [13]. Yeast also has biological functions, including effects on immune response dynamics [14,15,16]. One known constituent of yeast is beta-glucan, a component of yeast cell walls, which modulates innate immune functions through increasing phagocytosis, bactericidal killing, and oxidative burst of immune cells [17,18,19]. Thus, administration of a component of yeast cell walls enhances anti-bacterial immunity mediated by innate immune cells, including macrophages and neutrophils.

Several studies have reported that weaning has negative effects on the immune response of calves [4,11], but the effects of hydrolyzed yeast (HY) on immune and stress responses during weaning have yet to be explored. Therefore, the purpose of this study was to determine whether HY administration enhances the innate immunity of Holstein calves during weaning and alleviates weaning stress. Accordingly, the effects of HY supplementation (0.2%) on growth performance, health status, and innate immune parameters (e.g., pro-inflammatory cytokines, acute-phase proteins (APPs), and immune cell populations) were evaluated in weaning Holstein calves.

## 2. Materials and Methods

All animal experiments were conducted at the Dairy Science Division of the National Institute of Animal Science, Korea. The experimental procedures were reviewed and approved by the Ethics Committee of the Use of Animals in Research of the National Livestock Research Institute, Korea (approval number: NIAS-2019108).

### 2.1. Animals and Treatments

After separation from their mother, Holstein calves were moved into individual indoor pens and weighed. Each pen had solid iron rod sides, with openings for free access to food. Colostrum was given at 10% of body weight (BW) for the first 3 days. All animals were fed whole milk via a 2 L calf bottle, according to the step-down milking method [20]. Briefly, milk was provided up to 20% of BW until 28 days of age and then gradually reduced to 10% of BW between days 29 and 30, which was maintained until weaning (42 days of age). The calf starter and mixed grass hay (43% orchard grass, 43% tall fescue, and 14% white clover on a dry matter basis) were provided from days 7 and 28 onward, respectively. The timeframes for feeding and weaning are depicted in Supplementary Figure S1.

Eighteen calves were randomly allocated into two weaning groups, including CON (control; calf starter supplemented with no HY, *n* = 9) and HY (calf starter supplemented with 0.2% HY, *n* = 9). HY was provided with the calf starter from day 7 until the end of the experiment (49 days of age). Each group was provided with a similar nutritional quality (protein, 13.15 ± 0.34%; fat, 6.84 ± 0.21%; and lactose, 2.87 ± 0.04%). Chemical analyses of the diets were performed, and the percentages of the total protein, fat, and lactose in the colostrum were measured using a MilkoScan 104 apparatus (Foss Electric A/S, Hillerød, Denmark). The ingredients and chemical compositions of the calf starters are presented in Table 1. The experimental diets were sub-sampled (100 g) weekly and stored in a refrigerator at 4 °C. The collected sub-samples were pooled and used for the chemical composition analysis. To determine the DM content, the samples were dried in a forced air oven at 105 °C for 24 h and then crushed to pass through the 1-mm screen of a cutting mill (Shinmyung Electric Co., Ltd., Daegu, Korea) [21]. The protein content (N × 6.25) was determined according to the Kjeldahl method [21], using a DK 20 Heating Digester and Semi-Automatic Distillation Unit Model UDK 139 (VELP Scientifica, Usmate, Italy). The fat (HCl-fat) was extracted via diethyl ether after acid hydrolysis [22]. Both the CON and HY starter diets were formulated with identical nutrients, except that the HY treatment contained 0.2% HY (Progut™, Suomen Rehu Co. Ltd., Esbo, Finland) instead of soybean meal (SBM). The composition of Progut™ is 7–9% mannose, 10–12% beta-glucan, and 700–900 mg/kg of free mono-nucleotides.

### 2.2. Weaning and Blood Sampling

The calves were weaned at 42 days of age (0 days post-weaning (DPW)). To examine the changes in the immune- and stress-response-related parameters according to the weaning, blood was collected in 10-mL heparinized tubes (Vacutainer; BD, Plymouth, UK) from the jugular vein at 40 (−2 DPW), 43 (1 DPW), 45 (3 DPW), and 47 (5 DPW) days of age. Plasma was collected from the heparinized tubes after centrifugation at 1600× *g* for 15 min at 4 °C. The samples were stored at −80 °C until further analysis.

### 2.3. BW Gain and Feed Intake

We recorded the milk intake from 7 to 35 days of age. We recorded the calf starter intake, forage intake, and BW changes from 7 to 49 days of age. The average total dry matter intake (DMI; milk solids, calf starter, and forage) and feed efficiency (kg of BW gain/kg of total DMI) were also calculated.

### 2.4. General Health Monitoring

To monitor the overall health conditions of the experimental calves, we used respiratory and fecal scoring systems. The respiratory scoring was recorded on a 5-point scale: normal (1), slight cough (2), moderate cough (3), moderate to severe cough (4), or severe/chronic cough (5). Diarrhea severity was determined using the averages of fecal fluidity, consistency, and odor. Monitoring was conducted daily (08:00) for 1–2 h, in accordance with the method described by Larson et al.; [23]. Fecal fluidity was scored using a scale of normal (1), soft (2), runny (3), or watery (4); fecal consistency was scored using a scale of normal (1), foamy (2), mucous-like (3), sticky (4), or constipated (5); and fecal odor was scored using a scale of normal (1), slightly offensive (2), or highly offensive (3).

The calves received antibiotic treatments (sulfadimethoxine sodium, 55 mg/kg of BW daily; Green Cross Veterinary Products Co. Ltd., Yongin, Korea) when the fecal scores exceeded 3 for two consecutive days or when there were signs of severe disease (e.g., severe cough). Health scores were determined as an average of the diarrhea severity and respiratory scores. All health monitoring was conducted by investigators who were blind to the experimental treatments.

### 2.5. Hematology

Hematology was performed to determine the immune cell populations in the blood. The proportions of neutrophils (NE), lymphocytes (LY), and leukocytes were measured using an automatic analyzer (Hemavet 850; Drew Scientific, Portsmouth, RI, USA). The percentages (%) of neutrophils and lymphocytes and the number of leukocytes are presented.

### 2.6. Enzyme-Linked Immunosorbent Assay (ELISA)

The plasma concentrations of the APPs, transferrin and lactoferrin, and total serum immunoglobulin (Ig) were determined using ELISA kits (Bethyl Laboratory, Montgomery, TX, USA). The concentrations of haptoglobin (Hp; Life Diagnostics Inc., West Chester, PA, USA), cortisol (Oxford Biomedical Research Inc., Oxford, MI, USA), serum amyloid A (SAA; Tridelta Development Ltd., Maynooth, Co., Kildare, Ireland), and serum pro-inflammatory cytokines were also measured using ELISA kits according to the manufacturer’s instructions. Serum interleukin 1β (IL-1β) and interleukin 6 (IL-6) were assayed using the bovine cytokine screening set (Thermo Scientific, Rockford). Serum tumor necrosis factor α (TNF-α) and interferon γ (IFN-γ) were assayed using the DuoSet ELISA kit according to the manufacturer’s protocol (R&D Systems Inc., Minneapolis, MN, USA). The detection ranges were IL-1β: 125–2000 pg/mL, IL-6: 312.5–5000 pg/mL, TNF-α: 6.25–800 pg/mL, and IFN-γ: 78–10,000 pg/mL. The amounts of APPs and cytokines were measured using a microplate reader (Molecular Devices, Sunnyvale, CA, USA).

### 2.7. Flow Cytometry

To examine the proportions of CD4– and CD8-positive T cells in the blood, peripheral blood mononuclear cells (PBMCs) were separated from the whole blood buffy coat using a Ficoll-Paque™ plus (GE Healthcare, Piscataway, NJ, USA) density gradient. Briefly, whole blood was suspended in Ficoll-Paque™ plus and centrifuged at 1300× *g* for 20 min at 18 °C. The isolated cells were incubated for 20 min at 4 °C with monoclonal antibodies for CD4 or CD8. The cells were then washed three times with phosphate-buffered saline (PBS) and suspended in 0.2 mL of PBS. Flow cytometry was performed using a FACScanto flow cytometer (BD Biosciences, San Jose, CA, USA), and the data were analyzed using FACSDiva™ (BD Biosciences, San Jose, CA, USA).

### 2.8. Statistical Analysis

We performed a power analysis to determine the sample size using G*Power (version 3.1.9.4 for Windows, 1992–2019 Universität Kiel, Germany) as follows: (1) independent group *t*-test (significance level of 0.05, power of 0.8, and effect size of 1.5) and (2) repeated-measures analysis (significance level of 0.05, power of 0.8, effect size of 1.5, correlation among repeated measures of 0.5, and non-sphericity correction of 1.0). Based on the sample size test, the minimum group numbers required for (1) and (2) were nine and four calves, respectively. The power analysis followed the guidelines suggested by Cohen in the field of animal science [24]. The *post-hoc* compute-achieved power test to calculate the sample power of valid equations was conducted using the G*Power software (version 3.1.9.4) by adopting an error probability of 5% for the sample size used. The sample power (1-β err prob) was 0.85 (independent group *t*-test) and 1.0 (repeated-measures analysis).

All statistical analyses were carried out in SAS 9.2 [25]. Data concerning the serum cortisol, APPs, and pro-inflammatory cytokines were obtained for both groups (control and HY) at the four time points (−2, 1, 3, and 5 DPW). A repeated-measures ANOVA was performed to detect any differences in the groups according to the time interaction in addition to the main effects. A Bonferroni *post-hoc* test was performed for multiple comparisons. Data concerning the BW gain, feed intake (calf starter and forage intake), health monitoring (diarrheal incidence, diarrhea severity, and health score), ELISA assay (total serum Ig), hematology (NE, LY, NE:LY ratio, and leukocytes), and flow cytometry (CD4^+^ and CD8^+^) data were separately analyzed at each time point. Differences between the two groups at the same time point were determined using an independent group *t*-test. The results are presented as the mean ± standard error (SE), and the effects were considered significant at *p* < 0.05.

## 3. Results

### 3.1. Growth Performance and Feed Intake

The HY calf starter contained 0.2% dietary immune modulator instead of SBM, and the chemical compositions of the control and HY calf starter were similar (Table 1). The growth performance (BW gain and feed intake) of the calves fed different starters was evaluated before and after weaning. The calves fed the HY starter had a significantly-higher BW gain than the control-fed calves (*p* = 0.003; at 7 weeks of age, 4.11 ± 0.09 kg vs. 4.74 ± 0.05 kg; Table 2). There were no differences concerning the BW gain during the pre-weaning period, but the calves in the HY group consumed more calf starter (1–6 weeks of age, 2.59 ± 0.05 kg vs. 2.96 ± 0.03 kg, *p* = 0.001). The milk intake, total forage intake, total DMI, and feed efficiency were not affected by the dietary treatments.

### 3.2. Stress Hormone

**Figure 1:**
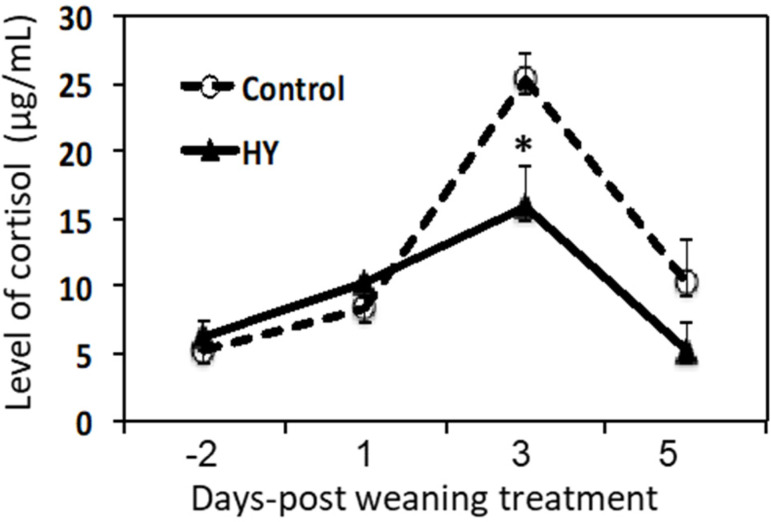
Changes in the serum cortisol levels of the calves fed the CON and HY calf starters after weaning. The dotted line represents the calves fed calf starter without HY (control diet (CON)) and the solid line represents the calves fed calf starter supplemented with hydrolyzed yeast (HY). Blood cortisol levels were assessed via an ELISA assay. The asterisk indicates a significant difference between the dietary groups (*p* < 0.05). Data are presented as the mean ± SE. Day 0 post-weaning represents the beginning of the weaning procedure (42 days of age).

Weaning influences the stress response [7], for which cortisol is a reliable stress marker in animals [8,9]. Weaning increased the cortisol concentrations in both groups. There were significant differences concerning the effects of the diet (*p* = 0.007), time (*p* = 0.0009), and the interaction between the two (*p* = 0.0006). Notably, the effects were more pronounced in the CON calves and significantly higher at 3 DPW (*p* < 0.001) (Figure 1).

### 3.3. Pro-Inflammatory Cytokines

**Figure 2:**
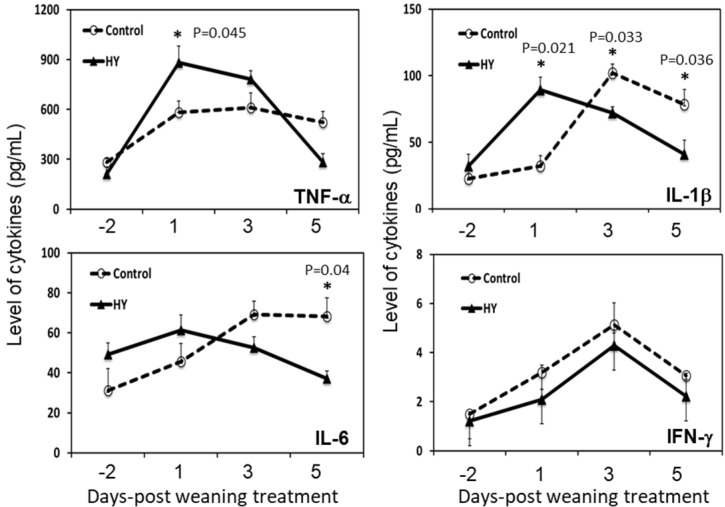
Effects of HY supplementation in calf starters on the changes in the levels of pro-inflammatory cytokines (TNF-α, IL-1β, IL-6, and IFN-γ) in calves after weaning. The dotted lines represent the calves fed calf starter without HY (control diet (CON)), and the solid lines represent the calves fed calf starter supplemented with hydrolyzed yeast (HY). The levels of pro-inflammatory cytokines in the blood were assessed via an ELISA assay. The asterisks indicate a significant difference between the dietary groups (*p* < 0.05). Data are presented as the mean ± SE. Day 0 post-weaning represents the beginning of the weaning procedure (42 days of age). TNF-α, tumor necrosis factor α; IL-1β, interleukin 1β; IL-6, interleukin 6; and IFN-γ, interferon γ.

Weaning influences immune–physiological responses in calves [26]. Therefore, the changes in the immune parameters and effects of the dietary treatments were measured. The results showed significant changes in the serum concentrations of the pro-inflammatory cytokines, TNF-α, IL-1β, and IL-6, after weaning (Figure 2). Before weaning, the calves in both groups had similar pro-inflammatory cytokine levels. However, after weaning, the calves fed the HY diet had elevated TNF-α and IL-6 levels (Figure 2). For example, the levels of TNF-α and IL-1β in the CON-fed calves were significantly lower than those of the HY-fed calves at 1 DPW (582.17 ± 67.0 vs. 882.12 ± 99.2 pg/mL (*p* = 0.045) and 32.17 ± 7.9 vs. 89.44 ± 9.5 pg/mL (*p* = 0.021), respectively). At 5 DPW, the HY-fed calves had significantly lower levels of TNF-α and IL-1β (*p* = 0.036). The IL-6 levels were similar at 1 DPW, whereas the IL-6 level in the HY group was significantly lower at 5 DPW (*p* = 0.04). There were no significant changes in the IFN-γ levels after weaning.

### 3.4. Acute-Phase Proteins

**Figure 3:**
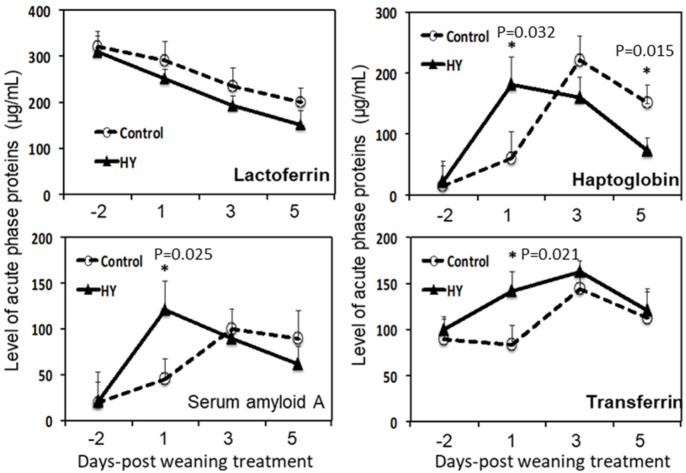
Effects of HY supplementation in calf starters on changes in the acute-phase protein levels in calves after weaning. The dotted lines represent the calves fed calf starter without HY (control diet (CON)), and the solid lines represent the calves fed calf starter supplemented with hydrolyzed yeast (HY). The levels of acute-phase proteins in the blood were assessed via an ELISA assay. The asterisks indicate significant differences between the dietary groups (*p* < 0.05). Data are presented as the mean ± SE. Day 0 post-weaning represents the beginning of the weaning procedure (42 days of age).

As the innate cytokines changed after weaning, other innate immune modulators were also investigated. Immune challenges, such as infections and stress-induced APP production as part of innate immunity, may regulate immune–physiological changes in animals. APP changes were monitored in the blood after weaning to evaluate whether the dietary treatments affected the APP response. The APPs, including Hp, SAA, and transferrin, increased after weaning (Figure 3). Interestingly, the APP patterns differed between the groups. The Hp (*p* = 0.032), SAA (*p* = 0.025), and transferrin (*p* = 0.021) levels were significantly higher in the HY group at 1 DPW. However, the elevated Hp levels in the HY-fed calves returned to the baseline levels earlier than those in the CON group at 5 DPW. Therefore, the APP levels of the CON group were significantly higher than those of the HY group at 3 DPW (*p* = 0.015).

### 3.5. Immunoglobulins

**Figure 4:**
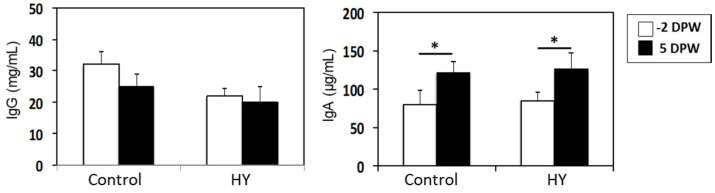
Changes in the serum IgG and IgA levels in the control- and HY calf starter-fed calves after weaning. The levels of immunoglobulins in the blood were assessed via an ELISA assay. The asterisks indicate significant differences between −2 and 5 DPW (*p* < 0.05). Data are presented as the mean ± SE. DPW, days post-weaning.

Changes in the serum immunoglobulins were monitored before and after weaning to evaluate whether the dietary treatments affected humoral immunity. The HY group had higher IgG levels at −2 DPW, but weaning did not induce changes in the IgG levels in either group. However, the IgA levels significantly increased after weaning in both groups, but there were no dietary effects (Figure 4).

### 3.6. Composition of PBMC

**Figure 5:**
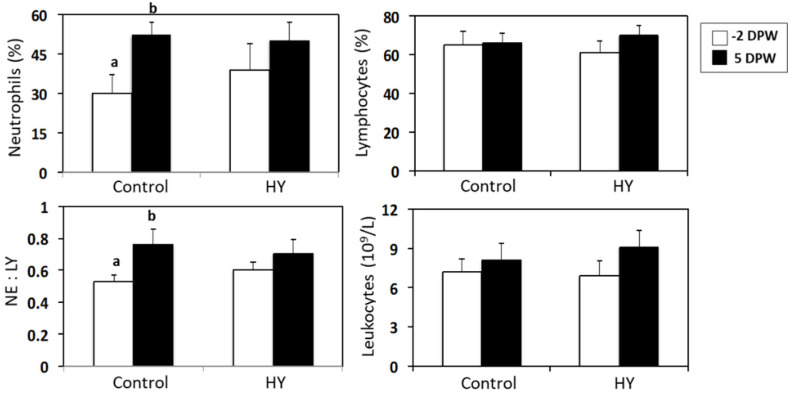
Changes in the composition and numbers of leukocytes in the calves fed different calf starters after weaning. Control represents calves fed calf starter without HY. HY represents calves fed calf starter with HY supplementation. Data are presented as the mean ± SE. Means with different letters differ significantly between −2 and 5 DPW (*p* < 0.05). NE, neutrophil; LY, lymphocyte; and DPW, days post-weaning.

**Figure 6:**
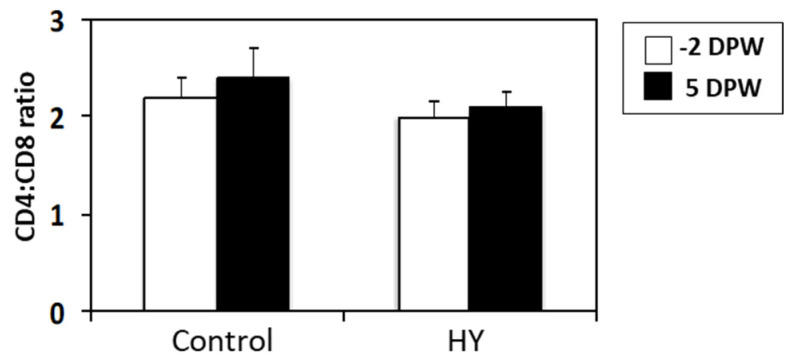
Changes in the ratio of the CD4^+^ and CD8^+^ T lymphocytes in the serum of the calves fed different starters during the pre- and post-weaning periods. Control represents calves fed calf starter without HY. HY represents calves fed calf starter with HY supplementation. The percentages of CD4^+^ and CD8^+^ cells among the lymphocyte gates were evaluated using flow cytometry. Data are presented as the mean ± SE. DPW, days post-weaning.

Weaning stress may affect the composition of immune cells in the peripheral blood. In this study, the non-weaned calves showed no changes in the peripheral blood composition (data not shown), whereas the weaned calves exhibited increased NE numbers in the PBMC. There were significant differences in the blood immune cell composition (NE and NE:LY ratio) of the CON and HY groups. As shown in Figure 5, the NE percentage increased significantly at 5 DPW (*p* = 0.007), which elevated the NE:LY ratio in the CON-fed calves (*p* = 0.012). The concentration of white blood cells did not differ before or after weaning. The percentage change (%) in the CD4^+^ and CD8^+^ T lymphocytes in the peripheral blood was investigated using flow cytometry, but there were no changes in CD4^+^, CD8^+^, or the CD4^+^:CD8^+^ ratio (Figure 6).

### 3.7. Health Status

To investigate the effects of HY supplementation on the health of calves before and after weaning, disease occurrence was monitored during the experiment. One calf in each group exhibited severe diarrhea symptoms and received antibiotics for two days. In general, the calves fed the HY diet maintained better health than the calves fed the control diet (Table 3). There were no differences in the health scores during the pre-weaning period, but the calves fed the HY diet had significantly lower diarrhea severity post-weaning than the CON-fed calves (*p* = 0.04) (2.53 ± 0.5 vs. 1.09 ± 0.2). Consistent with the milder diarrhea severity, the health score, which indicates disease severity, was significantly lower in the HY group. These results indicate that the HY group had a better health status than the control group after weaning.

## 4. Discussion

In animal husbandry, the calf-rearing period is important with regard to animal health and farm profitability. Therefore, the development of strategies to enhance the growth performance and health conditions of young animals are required. Physical and psychological stress events induce stress hormone secretion, which dampens immune cell activities. For example, weaning can increase the susceptibility of calves to infectious diseases owing to decreased immunocompetence. Dietary immune modulators can alleviate stress responses and strengthen host immunity, making them an effective strategy to improve the health conditions of weaned calves.

Several studies have investigated yeast-derived supplements (at levels between 0.001% and 2%) and have documented positive correlations with DMI, rumen pH, and nutrient digestibility [26,27,28,29,30,31,32,33,34]. In the present study, we used a 0.2% supplement level, which was sufficient to influence calf growth performance. Calves fed a HY diet had elevated BW gain during the post-weaning period and increased starter intake during the pre-weaning period. Similarly, previous studies have reported that calves fed yeast-supplemented diets showed increased BW gain [32,33]. There have been several reports of yeast products stimulating the growth of rumen microbes, which increases the initial digestion rate of cellulose-containing diets [35,36,37]. These reports suggest that the positive growth performance effects of HY may be attributed to the regulatory effects of HY on the rumen microflora. While we did not examine the changes in the rumen microbiome, it may be influenced by HY supplementation before and after weaning.

Weaning is a stressful process because of the shift from a milk-based to solid feed diet [38]. The concentration of glucocorticoids and cortisol in the blood is a reliable indicator of stress. In this study, cortisol increased after weaning (in both groups) but was more pronounced in the calves fed the control diet. This suggests that HY supplementation assists in the adaption of weaned calves to dietary changes.

As weaning stress dampens immunity [38], it is necessary to develop an effective dietary immune booster for weaned calves to prevent infectious disease. Yeast contains several functional ingredients that can affect an animal’s immune system [39,40]. Recently, several attempts have been made to use yeast-derived components as dietary immune modulators to improve animal health and immunity. A diet containing a 2% yeast culture improved the fecal scores and reduced the risk of health issues in Holstein calves [41,42]. In addition, the components of the yeast cell wall stimulate innate immune responses, such as macrophage activation [43,44]. In this study, the HY diet reduced diarrhea incidence after weaning, as observed in previous studies. Substances in the yeast cell wall, such as mannoproteins, beta-glucan, and oligosaccharides, may prevent mycotoxin adhesion to intestinal cell and reduce pathogen invasion [45]. These potential functional mechanisms are supported by the preferential adherence of pathogenic bacteria to the yeast cell wall [46]. We did not examine pathogenic bacteria or their adherence to the gut epithelium, but the reduced diarrhea incidence and improved health conditions may be explained by the direct regulation of HY products containing mannoproteins and beta-glucan. To fully understand the direct regulation of gut pathogens by HY products, it is necessary to investigate the changes in pathogen survival and adherence to the gut epithelium in HY-fed animals with intestinal pathogenic infections.

As discussed, yeast products can regulate host immune cells. Therefore, we investigated the innate and adaptive immune pathways in calves under weaning stress. The stress-induced secretion of pro-inflammatory cytokines regulates the secretion levels and/or stability of the hypothalamus–pituitary–adrenaline axis (i.e., corticotropin-releasing hormone and subsequent glucocorticoid secretion) [47,48]. Stress hormones dampen immune cell activity and increase susceptibility to infectious diseases. Furthermore, inflammatory cytokines are mediators of the immunological and pathological responses to stress and infection [49]. In this study, weaning induced the production of innate-type cytokines (TNF-α and IL-1β) in the serum of the calves, regardless of the diet. However, there were different dynamics concerning the innate cytokines in the blood of the calves from the two groups. The calves fed HY had significantly-higher levels of TNF-α and IL-1β compared to the CON-fed calves at 1 DPW. However, the cytokine levels dropped rapidly, returning to normal at 5 DPW (including IL-6). These results suggest that HY supplementation promotes pro-inflammatory cytokine production in response to weaning stress. Pro-inflammatory cytokines, IL-1 and IL-6, induce non-specific increases in the AAP concentrations of stressed animals (mainly cytokines) [50,51]. The APPs, Hp, and SAA in the HY supplementation group were significantly higher at 1 DPW. The APP levels quickly returned to normal at 3 DPW, mirroring the changes in the innate cytokines.

Transferrin, an iron-binding glycoprotein, was significantly increased in the HY group at 1 DPW. Transferrin plays a role in iron transport in the blood and is an indicator of the protein supply [52,53]. The increased level of transferrin at 1 DPW is consistent with the cortisol and pro-inflammatory cytokine results.

The arms of the adaptive immune system, T and B cell immunity, provide specific immunity against pathogens. Innate immunity is responsible for the efficient induction of adaptive immunity. We evaluated whether HY supplementation affects the adaptive immune response of weaning calves. Immunoglobulins are indicators of the humoral immune response [54]. Weaning increased the serum IgA levels, regardless of diet, indicating that weaning stimulates B cell immunity in the gastrointestinal tract. However, HY supplementation did not affect IgA production by the gut B cells. We examined the CD4- and CD8-positive T cell populations in the blood using flow cytometry, and, in line with the B cell results, we observed no differences in the T lymphocytes between the groups after weaning. HY supplementation may affect the functional activities of the T cells, such as cytokine expression, rather than the population size. Antigen challenge experiments are required to understand the effects of dietary immune modulators on adaptive immune responses under stress.

Various immune cells produce cytokines that serve as important mediators of communication in the immune system. Changes in the leukocyte population are a potential indicator of physiological stress and disease susceptibility in animals [55,56]. Anderson et al. [57] reported that the NE:LY ratio increased when cattle were challenged with dexamethasone. Previous studies also reported an increase in the NE:LY ratio in weaned calves [55] due to glucocorticoids [4], suggesting that glucocorticoids contribute to the NE:LY ratio. Many studies have reported that yeast or yeast products affect the composition of blood cells in weaned pigs or lipopolysaccharide-challenged piglets [13,58]. Nonnecke et al. [59] also reported that the total number of blood leukocytes and the composition of the mononuclear leukocyte population in neonatal calves were affected by the energy and protein intake. To gain a greater insight into the effects of yeast products on the blood cell composition of weaned calves, we conducted blood cell composition analyses. Interestingly, the CON-fed calves had an elevated NE:LY ratio due to an increase in NE. This pattern was not present in the HY-fed calves. These results suggest that the CON-fed calves were more stressed than the HY-fed calves.

## 5. Conclusions

Our study aimed to reduce the stress responses that occur in Holstein calves after weaning. Our results show that HY supplementation to the calf starter improves health conditions and alleviates stress responses. The HY diet facilitates the weaning-mediated innate immune response, including APPs and serum pro-inflammatory cytokines, which are necessary for resisting pathogenic infection. These results suggest that HY supplementation helps calves overcome dietary challenges by facilitating innate immunity and may increase resistance to stress. Therefore, weaned calves fed the HY diet may be more resistant to pathogenic infection. Nevertheless, our results do not fully explain the underlying mechanisms of these positive effects, nor is it clear how HY improves the health of Holstein calves. More in-depth studies of other factors, such as the intestinal microbiota and pathogen attachment to the gut epithelium, are required for a complete mechanistic overview of HY supplementation. This study contributes toward our understanding of the immune regulatory mechanisms of HY supplementation in weaned calves.

## Figures and Tables

**Table 1:** Ingredients and nutrient composition (%) of the control and hydrolyzed yeast (HY) calf starters.

Ingredient (% of DM)	CON	HY
Ground corn	19.98	19.98
Ground wheat	14.30	14.30
Molasses	5.00	5.00
Wheat hulls	15.00	15.00
Gluten feed	10.00	10.00
Soybean hulls	7.00	7.00
SBM	15.43	15.23
HY	−	0.20
Corn germ meal	5.00	5.00
Copra meal	5.00	5.00
Salt	0.50	0.50
Ca_3_(PO_4_)_2_	0.38	0.38
CaCo_3_	1.50	1.50
Bio–Zn	0.01	0.01
Virginiamycin	0.10	0.10
Premix (vitamins & trace minerals) ^1^	0.30	0.30
NaHCo_3_	0.50	0.50
Chemical composition (% of feed)	CON	HY
CP	17.50	17.52
CF	2.68	2.66
FAT	2.55	2.57
Ash	6.44	6.44

^1^ Premix supplied the following nutrients per kg of mixed feed: vitamin A, 4400 IU; vitamin D, 7331; vitamin E, 111 U; CuSO_4_.5H_2_O, 85.73 mg; zinc oxide, 55 mg; MnO_2_.H_2_O, 55 mg; and MgO, 1.86 g. CON, control; HY, hydrolyzed yeast; DM, dry matter; SBM, soybean meal; CP, crude protein; and CF, crude fiber.

**Table 2:** Growth performance, feed (calf starter, milk, and forage) intake, total DMI, and feed efficiency of the calves fed the CON and HY calf starters (mean ± SE).

	Calf Starter		
Variables	CON	HY	*p*-Value
BW gain (kg)			
Pre-weaning ^1^	11.58 ± 2.1	9.68 ± 1.3	0.11
Post-weaning ^2^	4.11 ± 0.09 ^a^	4.74 ± 0.05 ^b^	0.003
Calf starter intake (kg/week)			
Pre-weaning	2.59 ± 0.05 ^a^	2.96 ± 0.030 ^b^	0.001
Post-weaning	3.43 ± 0.05	3.31 ± 0.04	0.16
Milk intake (L/week)			
2–3 weeks	68.03 ± 1.4	71.80 ± 1.6	0.055
4–5 weeks	48.30 ± 0.3	47.00 ± 0.8	0.61
Total forage intake (g)			
Pre-weaning	510.41 ± 12.1	508.68 ± 10.3	0.66
Post-weaning	483.11 ± 13.5	447.35 ± 11.0	0.56
Total DMI ^3^ (kg)	47.08 ± 2.1	45.03 ± 1.1	0.47
Feed efficiency ^4^	0.43 ± 0.02	0.44 ± 0.01	0.56

CON = control; HY = hydrolyzed yeast; and DMI = dry matter intake. All calves were weaned at 6 weeks of age. ^1^ Pre-weaning = 1–6 weeks. ^2^ Post-weaning = 7 weeks. ^3^ Total DMI = milk solid, starter, and forage DMI during the whole experimental period. ^4^ Feed efficiency = kg of BW gain/kg of total DMI. ^a,b^ Means with different letters differ significantly between the CON and HY groups (*p* < 0.05).

**Table 3:** Diarrhea incidence, diarrhea severity, and health status of the calves fed the CON and HY calf starters before and after weaning (mean ± SE).

Variables	Calf Starter		*p*-Value
	CON	HY	
Diarrheal incidence ^1^ (%)			
Pre-weaning	11.11 ± 4.1	7.04 ± 3.1	0.22
Post-weaning	6.92 ± 2.2	3.18 ± 1.1	0.35
Diarrhea severity ^2^			
Pre-weaning	2.42 ± 0.7	2.33 ± 0.7	0.53
Post-weaning	2.51 ± 0.5 ^a^	1.09 ± 0.2 ^b^	0.04
Health score ^3^			
Pre-weaning	2.91 ± 1.3	2.61 ± 0.5	0.17
Post-weaning	2.76 ± 0.4 ^a^	1.11 ± 0.2 ^b^	0.03

CON = control; and HY = hydrolyzed yeast. ^1^ Diarrheal incidence (%) = proportion of animal(s) with diarrhea symptoms in the total animals of each experimental group on each day. ^2^ Diarrhea severity = averages of the fecal fluidity, consistency, and color. ^3^ Health scores = averages of the diarrhea severity and respiratory disease scores. ^a,b^ Means with different letters differ significantly between the CON and HY groups (*p* < 0.05).

## Supplementary Materials

The following are available online at https://www.mdpi.com/2076-2615/10/9/1468/s1, Figure S1: Feeding and weaning timeframes used in this experiment.
