# Supplementary figures and images for: Hydrolyzed Yeast Supplementation in Calf Starter Promotes Innate Immune Responses in Holstein Calves under Weaning Stress Condition
Source: Animals (Basel). 2020 Aug 21;10(9):1468. doi: 10.3390/ani10091468 (PMC7552225; doi:10.3390/ani10091468)

## SUPPLEMENTARY MATERIALS

Figure S1:

Feeding and weaning timeframes used in this experiment.

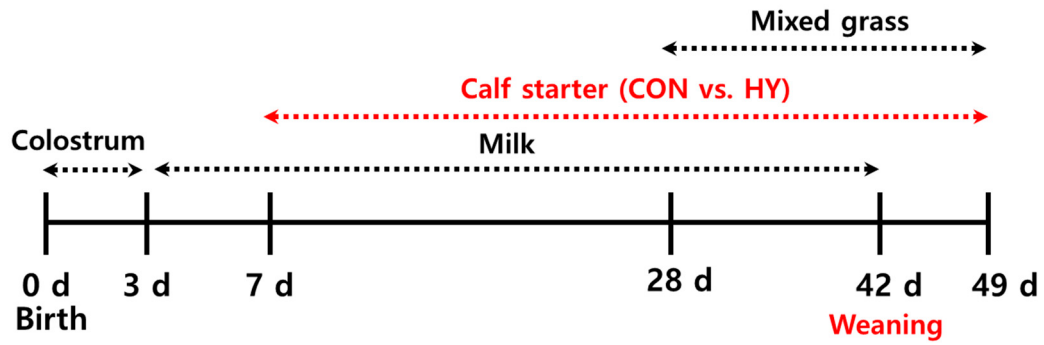

Supplement: Supplementary file 1 [file animals-10-01468-s001.pdf]
